# How Psychological and Behavioral Team States Change during Positive and Negative Momentum

**DOI:** 10.1371/journal.pone.0097887

**Published:** 2014-05-16

**Authors:** Ruud J. R. Den Hartigh, Christophe Gernigon, Nico W. Van Yperen, Ludovic Marin, Paul L. C. Van Geert

**Affiliations:** 1 Department of Psychology, University of Groningen, Groningen, The Netherlands; 2 Epsylon Laboratory, Faculty of Sport and Physical Education Sciences, Montpellier 1 University, Montpellier, France; 3 Movement to Health Laboratory (M2H), Faculty of Sport and Physical Education Sciences, Montpellier 1 University, Montpellier, France; Universidad de Zarazoga, Spain

## Abstract

In business and sports, teams often experience periods of positive and negative momentum while pursuing their goals. However, researchers have not yet been able to provide insights into *how* psychological and behavioral states actually change during positive and negative team momentum. In the current study we aimed to provide these insights by introducing an experimental dynamical research design. Rowing pairs had to compete against a virtual opponent on rowing ergometers, while a screen in front of the team broadcasted the ongoing race. The race was manipulated so that the team’s rowing avatar gradually progressed (positive momentum) or regressed (negative momentum) in relation to the victory. The participants responded verbally to collective efficacy and task cohesion items appearing on the screen each minute. In addition, effort exertion and interpersonal coordination were continuously measured. Our results showed negative psychological changes (perceptions of collective efficacy and task cohesion) during negative team momentum, which were stronger than the positive changes during positive team momentum. Moreover, teams’ exerted efforts rapidly decreased during negative momentum, whereas positive momentum accompanied a more variable and adaptive sequence of effort exertion. Finally, the interpersonal coordination was worse during negative momentum than during positive momentum. These results provide the first empirical insights into actual team momentum dynamics, and demonstrate how a dynamical research approach significantly contributes to current knowledge on psychological and behavioral processes.

## Introduction

During the 34th America’s cup (September 2013), the American catamaran came back from a 1–8 disadvantage to 8–8. Then, in the winner-takes-all deciding race, Team USA started lagging behind Team New-Zealand, but turned the momentum and sailed to a historical victory. While in the ancient Greek times Homer suggested that momentum shifts are controlled by Gods’ interference in human affairs (see [1]), current researchers acknowledge that positive momentum–progressing in relation to the goal–and negative momentum–regressing in relation to the goal–elicit psychological and behavioral changes, termed *psychological momentum* (PM) [2]. Still, researchers have not yet been able to capture *how* psychological and behavioral states actually change when teams acquire positive or negative momentum. In the current study we propose a paradigm advocated by dynamical systems theorists (e.g., [3], [4]), allowing us to experimentally examine changes in psychological and behavioral performance variables during positive and negative momentum.

### Earlier Research on Team Momentum

Periods of positive and negative momentum can be observed in various achievement contexts, such as presidential campaigns and business, but are probably most apparent in sports [1], [5], [6]. Hence, most research on team momentum has been conducted in this domain. Quantitative studies conducted so far have increased insights into *which* psychological variables are higher as a result of positive momentum, compared to negative momentum or no momentum [7]–[9]. For example, providing members of volleyball teams with questionnaires containing either a hypothetical positive momentum scenario (their team came back from behind) or a no-momentum scenario (the score kept close), researchers found that participants in the positive momentum scenario reported more momentum, confidence, and control, but also lower levels of anxiety and discouragement than participants in the no-momentum condition [7], [8]. Moreover, effects of the positive momentum scenario were found to be stronger if the momentum occurred in a crucial phase of the competition [8] and if the team members felt highly cohesive [7].

In an experimental study that took into account negative momentum as well, volleyball teams had to perform three competitive trials [9]. After each trial the experimenter indicated whether the team performed better (positive momentum condition) or worse (negative momentum condition) than the opponent team. The authors found that momentum perceptions, collective efficacy–team members’ perceptions of their team’s ability to successfully perform the task [10]–and positive affect were higher in the positive momentum condition, whereas negative affect was higher in the negative momentum condition. In line with this, perceptions of momentum and collective efficacy generally increased over the three positive momentum trials, while negative affect decreased. In contrast, momentum perceptions, collective efficacy, and positive affect decreased over the negative momentum trials, whereas negative affect increased.

These previous studies showed that positive team momentum leads to various positive feelings and perceptions, and negative momentum to negative feelings and perceptions. However, it remains a question *how* the psychological changes occur over the course of positive and negative momentum. Furthermore, it is unclear how momentum relates to performance change, because studies investigating the momentum-performance relationship have revealed mixed results. That is, researchers have suggested that performance improves with positive momentum [8], but several studies did not find this effect [8], [9], [11]. Likewise, negative momentum is typically assumed to result in performance deterioration [9], [11], but has also been linked to performance improvement [9]. This positive effect of negative momentum has been explained in terms of a negative facilitation tendency [12], [13], or in terms of reactance (see [14] in individual sports). According to both explanations, team members (or individual athletes) would increase their efforts after failure to overcome their negative momentum.

Taken together, previous research has demonstrated that both psychological variables and performance are influenced by momentum (positive or negative), but it remains unknown *how* these variables change over time. This could be attributed to the primary focus on snapshot measures after manipulated or naturally occurring momentum periods during a fixed time (or scoring) span. That is, team momentum studies examined psychological variables and performance outcomes at only one point in time (for an exception, see [9]; in this study measures were taken after three volleyball tasks, however, this does not allow for a true analysis of trajectories of psychological and performance changes). We therefore conducted a multidisciplinary process-oriented study aimed to provide the first insights into the nature of psychological and behavioral performance changes during positive and negative team momentum. These aims are in direct accordance with early [1] and recent [2] theoretical propositions stating that PM is a dynamical phenomenon.

### The Dynamical Nature of Team PM

According to early theoretical assumptions, positive and negative (team) PM states can emerge and disappear, and their intensity may increase or decrease [1], [15]. Based on qualitative results in handball, researchers recently suggested that positive and negative team PM involve multiple psychological (e.g., emotions, feelings of confidence and cohesiveness) and behavioral (e.g., level of energy and activity) factors, that both undergo upward and downward changes over time [16]. This suggestion supports the most recent theoretical definition of PM as “a positive or negative dynamics of cognitive, affective, motivational, physiological, and behavioral responses (and their couplings) to the perception of movement toward or away from either an appetitive or aversive outcome” ([2], p. 397). Gernigon and colleagues proposed that PM can be conceived as a dynamical system [2].

Simply put, a dynamical system is a set of interconnected elements that undergoes change [17]. According to the dynamical systems perspective, the state of a system does not merely vary as a function of the value of one or a few independent variables, but also as a function of its preceding states [18], [19]. That is, an event may change the state of a system (or not), depending on the history of the states of that system. Related to this, the change in the system’s state can be nonlinear [17], [20]. For instance, when the system finds itself in a stable negative state–e.g., being desperate after some errors–, one or a few positive events such as experiences of success may not directly boost one’s PM. On the other hand, when the stability of the system’s negative state is low–e.g., making errors, but knowing your form is not bad–, one positive event can be sufficient to give rise to a positive PM experience (for more theoretical explanations of the dynamical systems approach in psychological and social sciences, see [17], [20]–[22]). In individual sports, indications that PM can indeed be considered as a dynamical phenomenon have recently been found. In a qualitative study researchers found that positive and negative PM experiences involve a complex interplay between perceptions, emotions, cognitions, and behaviors [14]. Furthermore, in a recent experiment in which cyclists were competing, it was found that progressing in relation to the goal (i.e., victory) gives rise to a positive PM experience that develops relatively late, whereas a negative PM experience develops rapidly when regressing in relation to the goal [23].

### Examining Team PM Dynamics

The conception of team PM as a dynamical phenomenon [1], [16] and the analogy between PM and dynamical systems, implies that the dynamical systems theory (DST)–”an approach to the description and explanation of change” ([19], p. 243)–should be used to study this topic. Because it is impossible to measure all variables related to changes in team PM (these are numerous, see [16], [24]), an important step in obtaining an understanding is to track the development of global level variables that can best describe team PM (see [17], [25]). Literature on team performance considers collective efficacy as a crucial global team variable, which is related to team momentum and may dynamically fluctuate over time [9], [10]. Indeed, one earlier study already found a general increase in collective efficacy in a positive momentum scenario and a decrease in a negative momentum scenario [9], which is in line with the suggestion that teams can enter a positive and negative efficacy-momentum spiral during a competition [10].

Another global psychological team variable is task cohesion, which is the degree to which team members work together to achieve a task or goal [26]. Task cohesion is considered a powerful team attribute highly related to performance [27], [28]. Moreover, it is considered a dynamical construct, which may vary from second to second during a competition [29] and is related to team momentum [1], [7]. Positive and negative dynamics in both team efficacy and task cohesion may thus reflect the development of positive and negative team PM experiences.

The ongoing performance process during positive and negative team momentum has not yet been empirically studied. As discussed earlier, research has mainly focused on performance outcome measures of momentum (e.g., [8], [9]). However, the earliest theoretical work on momentum already suggested that the performance process in terms of effort exertion undergoes typical changes over the course of positive and negative momentum [1]. More specifically, according to Adler’s theory, the start of positive momentum can be characterized by momentum building, a phase in which efforts are high. Once momentum is established, a phase characterized by an economy of efforts or “cruising” would be observed, during which a moderately strong level of exertion is sustained. With the goal within reach, effort exertion may naturally decrease, called “coasting” (see also [14]). Then, as the goal to be reached is near, a “re-momentum” is common, during which more efforts are exerted than previously, as a kind of “kick towards the finish”. Next to this dynamic development of effort exertion, the theory also states that positive momentum accompanies high coordination and rhythmicity of movements [1].

On the other hand, the performance tendency during negative momentum would generally be negative. However, at the start of the negative momentum period, a team may exert high efforts to overcome this (for a comparable tendency in individual momentum, see [13], [14]), which carries the risk of an overabundance of efforts [1]. Subsequently, voluntarily abandoning the activity is a common response when the negative momentum persists. When this is impossible (e.g., during a sports match), people may continue sinking until the end of the activity. Furthermore, movements would be more erratic during negative momentum [1].

Thus, based on the earlier literature on team momentum, we considered collective efficacy, task cohesion, exerted efforts, and interpersonal coordination as crucial performance, and team PM-related variables that may display specific dynamics during positive and negative momentum. To provide a first empirical examination of the dynamics involved in team PM, we used a rigorous experimental dynamical systems method, originally intended to experimentally study how different coordination patterns form in biological systems [3], [4]. According to this method, a parameter (i.e., control parameter) should be scaled upwards and downwards to examine how the system moves to its different collective states. Given that positive and negative PM develop when progressing or regressing in relation to the goal, experimentally scaling a team’s progress and regress would allow a thorough examination of the psychological and behavioral team dynamics during positive and negative momentum. For the current study, team rowing was chosen as a research context, because team members are highly interdependent in this type of sport, both psychologically and behaviorally. In addition, objective measures of force exertion and interpersonal coordination could directly be obtained in an experimental setting (i.e., on rowing ergometers).

In the remainder of the article we aim to provide the first empirical insights into how team members’ psychological states (collective efficacy and task cohesion) and behaviors (effort exertion and interpersonal coordination) change during positive and negative momentum. We will show that–as could be expected when considering team PM as a dynamical phenomenon–the values of psychological and behavioral states are not simply high or low during positive and negative momentum. Rather, the psychological and behavioral temporal patterns we found appear to closely follow the earliest social theory of momentum [1].

## Materials and Methods

### Ethics Statement

**Figure 1:**
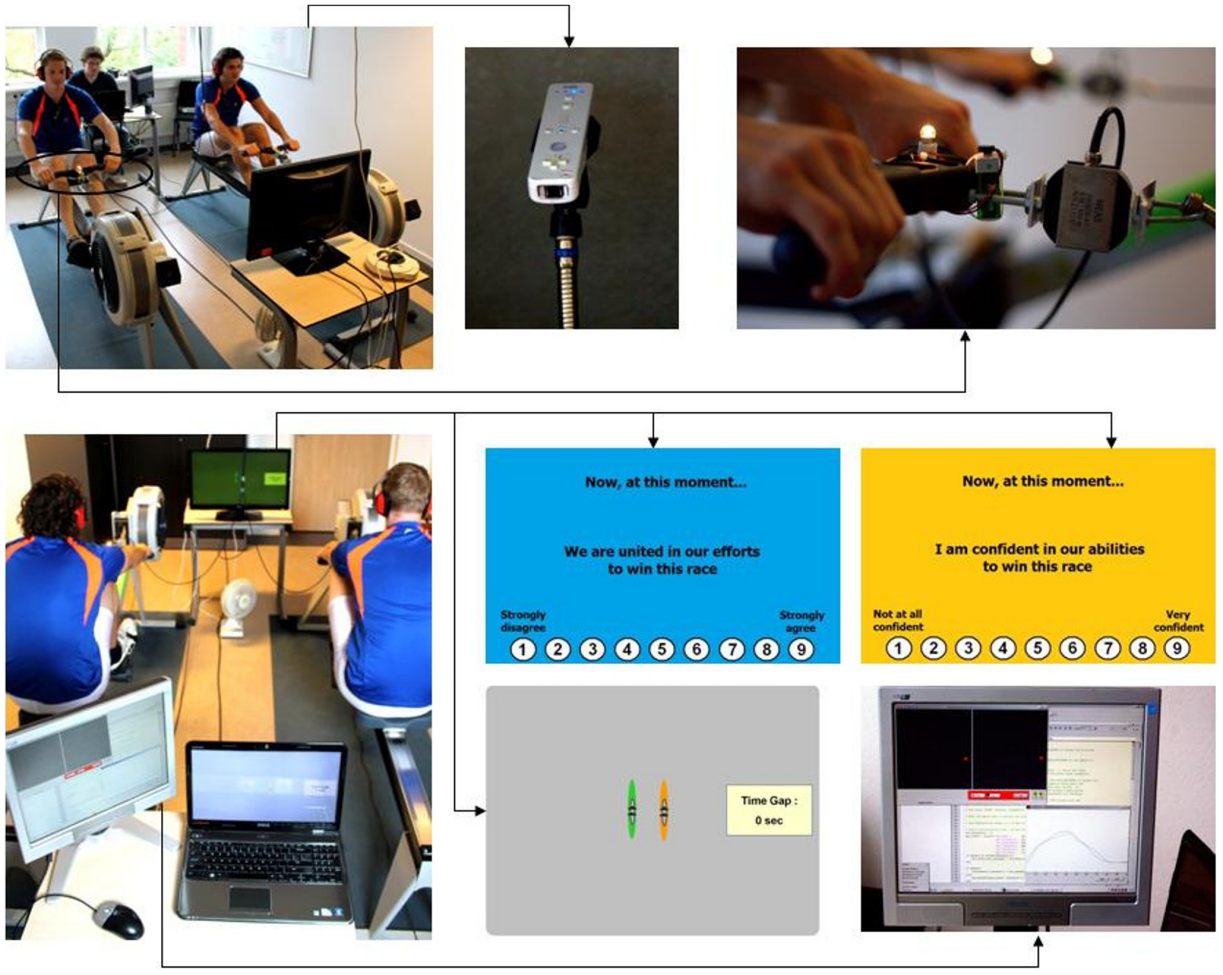
Research setup.

To demonstrate our research setup (displayed in Figure 1), a photographer took photos including two individuals. The two individuals have given their written informed consent (as outlined in the PLOS consent form) to publish their details. These individuals were not taking part in the actual study.

Our study protocol was approved by the Ethical Committee of the Department of Psychology, University of Groningen. The participants involved, who had orally consented to participate before the study, also provided their written consent when they arrived in the experiment room for their first rowing session. Although all participants were healthy competitive rowers, they also filled out a medical health form as an additional check. None of the participants indicated any (history of) medical problems before the start of the study. Finally, to hide the study aim for the duration of the study, participants were debriefed by e-mail when they all had finished.

### Participants

To optimize the validity of our design and the resulting outcomes, we recruited a sample consisting of participants for whom reaching a goal during a rowing task would be meaningful. Hence, we contacted a board member of a rowing club to approach competitive rowers. Twenty-two Dutch rowers (18 male and 4 female) of four different rowing teams participated. Their mean age was 20.14 years (*SD* = 1.86), and on average the participants were active rowers at a rowing club for 1.14 years (*SD* = 1.02). All four teams practiced together several times a week for about five months. In the current study, eleven teams of two rowers were formed by pairing the participants randomly with one team member.

### Experimental Design

The study took place in a room of the university, in which a research setup was built for this study (see Figure 1). The setup included two rowing ergometers (Concept 2, Model E, Inc., Morrisville, VT), a table with a monitor in front of the ergometers, a table with two computers behind the ergometers, Nintendo Wii remotes above the ergometers, and force sensors (MEAS, France) attached between the handles and the chains of the ergometers. On both ergometers we set the drag factor at 120 with PM4 performance monitors. This drag factor value corresponds to the resistance set by rowers for their workouts. While one of the computers behind the ergometers served to register the data from the Wii remotes and force sensors (see measures section), the other computer served to create the positive and negative momentum scenarios with race simulation software. This software enabled to program races involving (moving) avatars of two rowing boats that could be displayed on the screen in front of the ergometers. Furthermore, the software allowed entering items (i.e., questions the participants had to answer) at fixed intervals during the race.

#### Race scenarios

**Figure 2:**
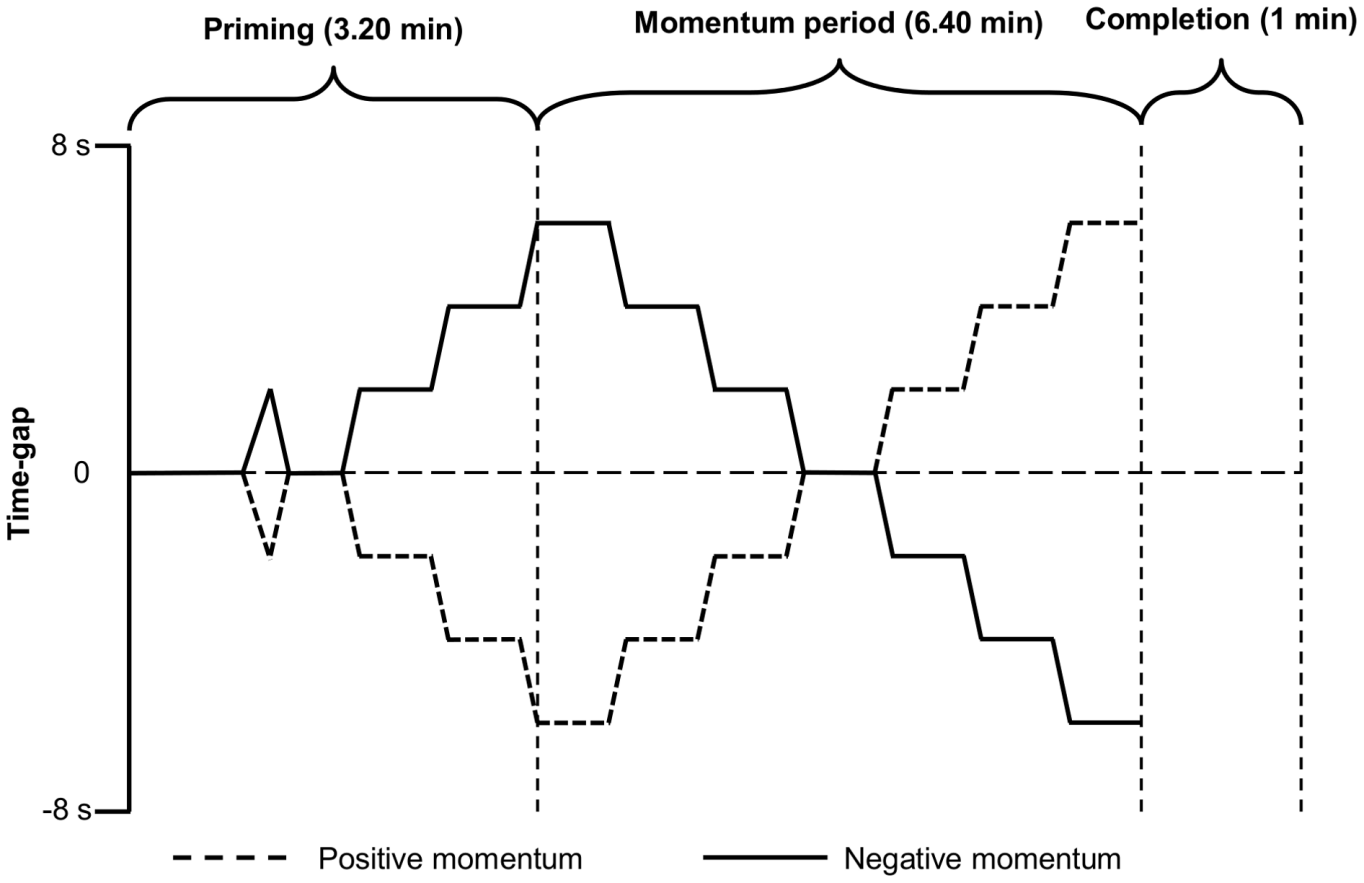
Illustration of the constructed positive and negative momentum scenarios.

To program credible race scenarios for our participants, we constructed the races in consultation with (inter)national rowers and rowing coaches, and we pilot tested some scenarios with four rowers and four other athletes in eight sessions. When rowing against an opponent of comparable level, a time-gap of 8 seconds was perceived as considerable, but doable, while more than 8 seconds would become unrealistic. The maximum duration of a strenuous rowing exercise turned out to be between 10 and 13 minutes. Taking this information into account, we programmed momentum scenarios that followed the experimental guidelines as set by earlier researchers [3], and included three phases: A priming phase, a momentum phase, and a completion phase (see Figure 2).

The priming phase covered the first 3.20 min. During the start of this phase, the boats on the screen kept in step and one of the boats took a short lead to add credibility to the scenario. Then, the boat of the participants either moved to a lag of 6 seconds, or to a lead of 6 seconds, which was the starting point for the positive or negative momentum phase, respectively. During the momentum phase that followed, the team’s boat gained 2 seconds each minute until leading by 6 seconds (positive momentum), or lost 2 seconds each minute until lagging behind by 6 seconds (negative momentum). This phase lasted 6.40 minutes. During the completion phase, which lasted 1 minute, the final time-gap between the boats was reached, which was between a 6-second lag and a 6-second lead. This phase was not included in the data analyses, but was added to avoid participants thinking that they were involved in identical race scenarios (although they were kindly requested not to discuss their race with other participants). However, none of the races ended in a (full) victory or defeat for the participating teams (i.e., winning or losing by 8 seconds).

In addition, while the changes in configuration were displayed on the screen during the entire race, the elapsed time was not. Hence, the display *only* included progress and regress in relation to the goal of gaining 8 seconds. This meant that, from the participants’ perspectives, the goal of gaining 8 seconds could occur at any time, and gaining 2 seconds brought the team closer to the goal, whereas losing 2 seconds brought the team further away from the goal. By doing this, we solved the experimentally problematic conflation between progress/regress in relation to the goal and the distance from the outcome [2], [30], [31].

### Procedure

Each team (pair) participated in two sessions–one positive momentum session and one negative momentum session–in random order, spread over one to two weeks. Upon their arrival, we gave the participants a quick tour through the experiment room, during which we showed how we were able to capture their exerted efforts and coordination, and explained that we could connect their real-time performance to racing software. This tour served to avoid suspicion about possible manipulations during the study, and preceded the participants’ warm-up activities. After the warm-up, we explained to the participants that they would be connected to the racing software. We told them that the race would be displayed on the screen in front of them, and we provided them with a clear goal: To beat the opponent by taking an 8-second lead. We added that if the race would become too long, it would be stopped to avoid too much exhaustion (note that in reality the race was already programmed at 11 min with no ultimate winner).

We explained to the participants that they would see two boats on the race screen, a green and an orange boat. The green boat represented the participants’ boat, whose speed would be based on a combination of their shared effort exertion and their coordination, as continuously collected by the racing software. We said that the speed of the other boat was based on the performance of another team at a comparable level, whose data had already been collected and uploaded into the software. Furthermore, we told the participants that the screen changed regularly to display two questions, and that the race screen would be shown again when both participants had verbally answered the questions. To avoid participants being able to hear each other and be influenced by each other’s item answers during the race, we gave them soundproofed headsets. The participants’ answers were recorded by voice recorders attached to their t-shirts.

When the participants were ready, a research assistant counted down and the race, along with the data collection from the force sensors and the Nintendo Wii remotes were started. While they were rowing, the participants followed the (manipulated) development of the race on the screen. After the second session, the participants were asked to fill out a questionnaire including a manipulation check. All participants indicated a period corresponding to the actually manipulated momentum phases in their answers to the questions: “Was there a period you were moving toward the victory?”, “Was there a period you were moving toward the defeat?”, and “if yes, when was this period?”.

### Measures

#### Psychological variables

To minimize the possible interfering influence of answering questions during the race, we only picked one collective efficacy item and one task cohesion item, which could be verbally answered on a 9-point scale while rowing. The items appeared on the race screen 20 s after each change in time gap between the boats (i.e., each min). Collective efficacy items generally include team members’ confidence in their team’s abilities to produce specific attainments (e.g., bounce back from performing poorly) [32], [33]. Often, one general measure of collective efficacy is included in questionnaires as well, which reflects the team members’ confidence in the team’s abilities to win the competition, or outperform the other team [9], [33]. Therefore, we included such an item in the software, namely “Now, at this moment I am confident in our abilities to win this race” (1 = *not at all confident*, 9 = *very confident*).

A widely used cohesion questionnaire in achievement contexts, and in sport in particular, is the Group Environment Questionnaire (GEQ). We picked the item with the highest loading on the (group integration) task cohesion dimension found in a validation study of the questionnaire [34]. The original item is “The members of my team are united in their efforts to reach the performance goals”, which we adapted to our research context by formulating the item as “Now, at this moment we are united in our efforts to win this race” (1 = *strongly disagree*, 9 = *strongly agree*).

#### Performance variables

Pre-calibrated force sensors were attached between the handles and the chains of the ergometers to provide continuous data of effort exertion. The two force sensors were connected to a data acquisition card (DAQ), made by National Instruments (NI USB-6009). The DAQ served to transfer the data from the two force sensors to the computer via USB. A Matlab script was written to save that data in Volts (V) at a frequency of 100 Hz.

Nintendo Wii remotes, attached to the ceiling above the ergometers, contain infrared (IR) camera sensors (PixArt Imaging, Inc., Santa Clara, CA). The camera sensors tracked a light, which we placed on the handlebar of each ergometer, and which (also) emitted infrared light. This system provides accessible, high resolution and high-speed movement tracking [35]. The temporal accuracy of the IR camera sensors was 100 Hz. We determined the spatial accuracy of the sensors by putting a light (the same as those placed on the handles) on a big rotating record turntable, placed at the same height as the handlebar. As the light continuously visited the same coordinates during each rotation, the Nintendo Wii IR camera sensors measured each coordinate within an error margin of 0 to 2 millimeters. Given the length of a rowing stroke–about 150 centimeters–we considered a spatial accuracy of 2 millimeters (maximum) to be acceptable.

During the experiment, an application written in C allowed us to collect the (changing) positions of the lights in pixels (pix) via Bluetooth, while simultaneously collecting the exerted effort data.

### Analyses

Before analyzing the data, the responses to the psychological items collected with the voice recorders were (double) checked by research assistants and entered in Microsoft Excel. The mean scores of the two members of each team were used for the analyses. The data in V from the force sensors were transformed to Newton units (N) according to a linear transformation provided by the manufacturer of the sensors. The mean force exertions per team in N were then taken into account for the analyses.

The positions of the handle bars as tracked by the Wii remote IR cameras in pix were transformed to centimeters (cm). Subsequently, we used a Butterworth filter in Matlab on the two time series of the positions, with a cut-off frequency of 4 Hz. We standardized the time series signals, and with the following formula we calculated the continuous relative phase (φ) via a Hilbert transformation [36] to obtain accurate quantifications of the interpersonal coordination between the participants:
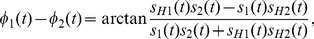
(1)where 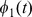 and 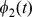 are the phases of each separate signal; 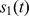 and 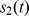 correspond to the real signals; and 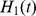 and 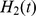 correspond to the Hilbert transformations of 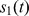 and 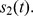


We then applied Monte Carlo permutation tests for the actual analyses. The Monte Carlo test determines the probability that an observed outcome is caused by chance alone, by simulating that chance [37], [38]. This is based on a repeated redistribution (e.g., 5000 times) of the collected data, to determine the possibility that a similar or more extreme result can be found by chance. A great advantage of this technique is that the test statistics are based on the observed data distribution, rather than on a presumed (normal) distribution. Therefore, this procedure often has better explanatory value than conventional statistical techniques in the field of behavioral and social sciences, such as ANOVAs, particularly in the case of smaller sample sizes and skewed data distributions [38]. In addition, the Monte Carlo technique is well suited to answer research questions that are difficult, or impossible to answer with conventional statistical techniques. One example is the calculation of a combined *p*-value, which we conducted for the mean relative phase and its standard deviation, in order to determine the quality of the coordination (see below).

Before running the Monte Carlo procedure, we divided the time series of the mean force exertion, the relative phase in degrees (φ), and the standard deviation of the relative phase (SDφ) into seven sections, corresponding to the seven periods in which there was a specific time-gap between the boats on the screen, and to the number of psychological measures. Subsequently, we ran the Monte Carlo procedure, for which we shuffled the data of the different variables within the teams (pairs), rather than over the entire sample. The reason for this was that different teams could not be considered as one homogeneous sample [25], [39]. This choice thus enabled us to find regularities in the team dynamics, despite the heterogeneity of the teams (e.g., some teams had more power than other teams, which could obscure the presence of dynamical trends in exerted efforts shared between teams). With the Monte Carlo procedure, the observed outcome was compared to the outcome of the redistributed data after each round of shuffling. In this way, we tested 1) the overall change in the variables during positive and negative momentum separately, 2) differences between the overall changes in the positive and negative momentum scenarios, 3) time-gap to time-gap differences in mean exerted force during positive and negative momentum, and 4) differences between the scenarios in terms of collective efficacy, task cohesion, and a combination of the mean relative phase (φ) and its standard deviation (SDφ). A low probability (*p*-value) that the randomly redistributed data generate the same, or more extreme, results than those actually observed indicates that the observed results are unlikely to be caused by chance alone. Finally, we estimated effect sizes by calculating Cohen’s *d* (observed result divided by the pooled SD) for each separate team result, and we reported the average effect size based on the individual team results.

## Results

Our results are based on the positive and negative momentum sessions of eight male teams (for an overview of the sample means and standard deviations of the psychological and behavioral variables, see Table 1). One team member of a female team mistakenly believed that, during the first session, her team was the orange boat, despite the instruction that they were the green boat. Furthermore, one female team and one male team literally gave up rowing during their (first) negative momentum session. The data of these three teams could therefore not be included in the analyses of the dynamics over both the positive and negative momentum session. Results of the psychological and behavioral dynamics will be reported separately.

**Table 1:** Overview of sample results (mean ± SD) for collective efficacy, task cohesion, exerted efforts, and relative phase according to momentum scenario and time-gap in seconds.

	Collective efficacy		Task cohesion		Exerted efforts (N)		Relative phase (°)	
Scenario:	Positive	Negative	Positive	Negative	Positive	Negative	Positive	Negative
**Time-gap**								
All	6.29±1.22	5.20±1.79	6.87±1.38	6.10±1.57	146.33±8.82	144.53±10.20	4.86±3.49	5.48±3.79
−6	4.44±.56	3.13±1.16	6.38±1.75	4.88±1.66	156.21±9.79	138.71±8.72	3.37±3.28	4.26±2.63
−4	5.63±.74	3.69±.84	6.25±1.31	4.88±1.46	147.20±7.90	139.68±7.00	4.58±4.53	5.70±3.50
−2	6.13±.58	4.38±.99	6.69±1.19	5.69±1.19	145.51±8.19	141.22±6.75	4.93±4.30	6.79±4.40
0	6.31±.70	5.25±.93	6.63±1.53	6.00±1.31	143.06±8.69	141.77±8.19	5.79±3.01	5.94±4.63
+2	6.75±.85	5.63±.64	7.06±1.12	6.56±1.08	144.20±6.15	143.91±7.86	4.21±3.41	5.67±3.79
+4	7.25±.71	6.38±.92	7.38±1.36	6.81±1.00	142.26±7.39	148.93±8.19	5.82±3.29	5.30±4.45
+6	7.56±1.05	7.94±1.15	7.69±1.22	7.88±1.06	145.85±8.25	157.48±12.24	5.30±3.04	4.71±3.72

Exerted efforts are expressed in Newton units (N), and relative phase measures in degrees (°). Time-gap is in seconds.

### Psychological Dynamics

**Figure 3:**
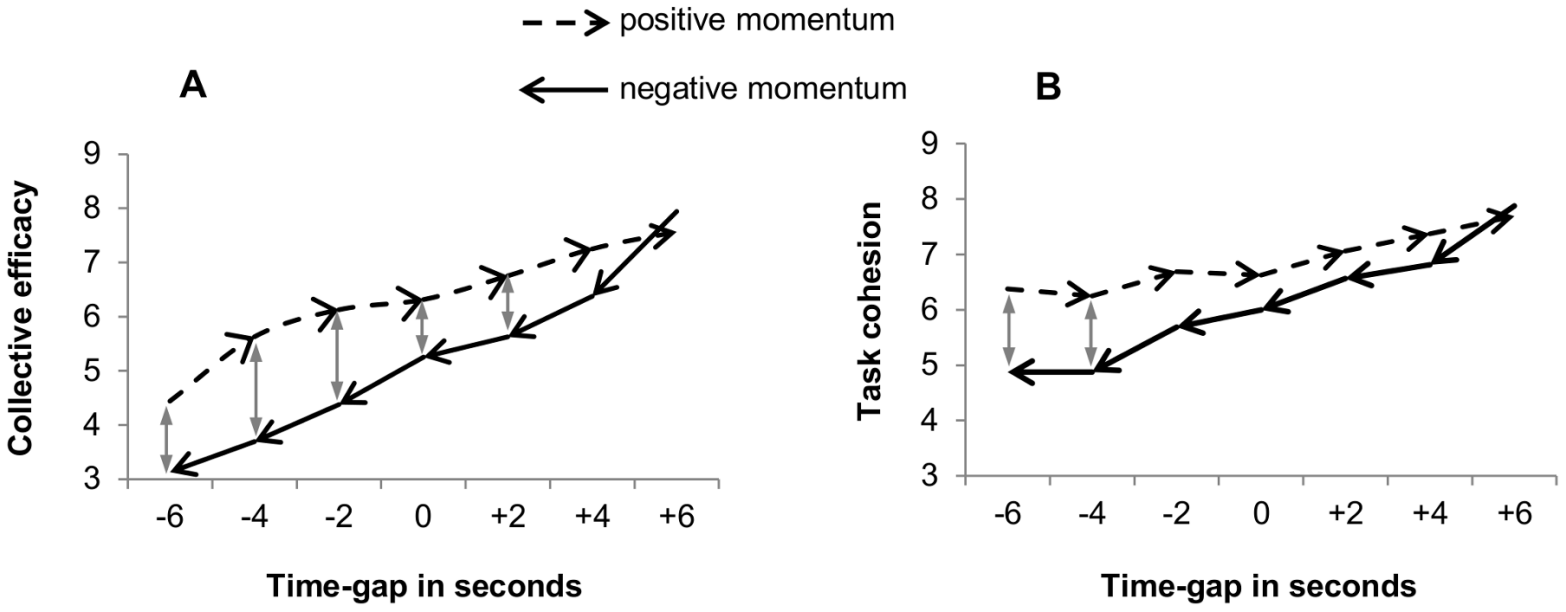
Dynamical trends of collective efficacy (A) and task cohesion (B) during positive and negative momentum. Grey double arrows indicate at which time-gaps there is a significant difference (p<.05) between the positive and negative momentum scenario.

Figure 3A shows the dynamics of collective efficacy. Monte Carlo tests revealed that this variable significantly increased during positive momentum (*p*<.0005, *d* = 7.12), and decreased during negative momentum (*p*<.0005, *d* = 6.74). The decrease during negative momentum was significantly steeper than the increase during positive momentum (*p*<.01, *d* = 2.25). In addition, collective efficacy was higher during positive momentum than during negative momentum (*p*<.001, *d* = .67). Significant differences (*p*<.05) between the scenarios were found at time gap values from −6 s until +2 s.

The dynamics of task cohesion are displayed in Figure 3B. This variable increased significantly during positive momentum (*p*<.0005, *d* = 2.73), and decreased significantly during negative momentum (*p*<.0005, *d* = 4.18). The decrease during negative momentum was significantly steeper than the increase during positive momentum (*p*<.01, *d* = 2.43). Moreover, task cohesion was generally higher during positive momentum than during negative momentum (*p*<.001, *d* = .75), and local differences were found at time gap −6 s and −4 s (*p*<.05).

### Behavioral Dynamics

**Figure 4:**
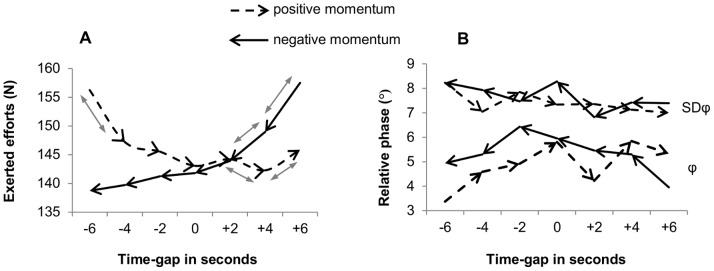
Dynamical trends of exerted efforts (A) and interpersonal coordination (B) during positive and negative momentum. Exerted efforts are expressed in Newton (N). The grey double arrows in Graph A indicate significant changes (p<.05) from time-gap to time-gap. The mean relative phase and its standard deviation (Graph B) are expressed in degrees.

Figure 4A displays the dynamics of exerted efforts. Based on the Monte Carlo tests we found that exerted efforts significantly decreased during positive momentum (*p*<.0005, *d* = 2.03) as well as negative momentum (*p*<.0005, *d* = 4.10). Overall, the decrease was steeper during negative momentum than during positive momentum (*p*<.01, *d* = 1.54). Accordingly, exerted efforts did not differ between scenarios at the start of the momentum periods–i.e., at +6 s in the negative momentum scenario and −6 in the positive momentum scenario–, whereas force exertion was significantly higher at the end of the positive momentum scenario–i.e., at +6 s–than at the end of the negative momentum scenario–i.e., −6 s–(*p*<.05).

Looking at the dynamics within the scenarios, pairwise comparisons between time gaps in the positive momentum scenario showed that effort exertion significantly *de*creased from time gap −6 s to −4 s and from time gap +2 s to +4 s (*p*<.05). A significant *in*crease in efforts was found from time gap +4 s to +6 s (*p*<.05). During negative momentum, significant *de*creases were found from time gap +6 s to +4 s and from +4 s to +2 s (*p*<.05).

The dynamics of the relative phase (φ) and its standard deviation (SDφ) are shown in Figure 4B. Overall, the combination of the mean continuous relative phase (φ) and its stability (SDφ) was better (i.e., closer to 0) over the course of positive momentum than during negative momentum (*p*_combined_ <.05). However, no significant differences were found between the scenarios at same values of time gaps. Within the scenarios separately, we found a decreasing trend in SDφ during positive momentum (*p* = .05, *d* = .69), which significantly differed from the slight increasing trend during negative momentum (*p*<.05, *d* = .98). Regarding the mean relative phase (φ), we found no significant increasing or decreasing trends during either positive or negative momentum.

## Discussion

Previous empirical research has demonstrated that psychological states and performance are often influenced by positive and negative team momentum [7]–[9]. Insights into the nature of psychological and behavioral performance changes during positive and negative team momentum are still lacking, however. To provide such insights, we applied a dynamical systems approach to examine psychological (collective efficacy and task cohesion) and behavioral (exerted efforts and interpersonal coordination) changes by experimentally varying progress and regress in relation to the team goal of winning the race (cf. [3]). This approach is in concordance with theoretical propositions stating that PM is a dynamical phenomenon [2], which, as we will discuss below, is supported by our data on the psychological, as well as the behavioral dynamics.

### Psychological Dynamics

With regard to collective efficacy, we found an increase during positive momentum and a decrease during negative momentum, which supports the theoretical assumption that teams may enter a positive or negative efficacy-momentum spiral during performance [10]. In addition, these results are in line with the earlier finding that team members’ collective efficacy increased and decreased when they experienced repeated success and failure, respectively [9].

A similar fluctuating pattern was observed for task cohesion: An increase was present during positive momentum and a decrease during negative momentum. In an earlier study, it was already found that task cohesion is related to team PM [7]. However, in that study the authors treated task cohesion as a “static” team attribute influencing the extent to which teams are sensitive to positive momentum periods, whereas the current study shows that task cohesion is also actually involved in the PM process. We therefore propose that task cohesion is a dynamical, fluctuating variable [29] that undergoes positive and negative changes during positive and negative momentum. All in all these results of collective efficacy and task cohesion suggest that the upward and downward dynamics of these variables characterize the psychological experience of positive and negative team PM, respectively.

Interestingly, the nature of the changes in collective efficacy and task cohesion was different depending on whether momentum was positive or negative. More specifically, decreases in collective efficacy and task cohesion during negative momentum were steeper than the increases during positive momentum. This asymmetry could not be detected in earlier snapshot studies on team momentum (e.g., [7], [8]), and was therefore not anticipated. Yet, the asymmetry supports the general assumption that negative events have a bigger psychological impact than equivalent positive events [40], [41]. Moreover, it is in line with results from individual sports, showing that negative PM was triggered more easily than positive PM [23]. Related to this asymmetry, collective efficacy and task cohesion were generally higher in the positive momentum scenario than in the negative momentum scenario. Given that the scenarios were exactly symmetrical, this finding suggests that team PM experiences are not only dependent on the static situation within the competition, but also on the history of progress or regress (cf. [2], [23], [42]). This thus implies that team PM is history dependent–a typical dynamical property–, which supports the proposition that PM could be considered a dynamical system [2].

It is noteworthy that the history of progress or regress particularly played a role when being behind (i.e., at negative values of time gap). This means that having gained the lead at the start of the race–the start of the negative momentum scenario–accompanied approximately the same levels of collective efficacy and task cohesion, as having gained the lead after being behind–end of the positive momentum scenario. On the other hand, lagging behind after having had the lead–end of the negative momentum scenario–accompanied lower collective efficacy and task cohesion than lagging behind at the start of the race–start of the positive momentum scenario. This suggests that in particular losing while having been close to the goal (i.e., winning) has a disproportionally strong psychological impact compared to losing while one has never been close to the goal. This finding is in accordance with the well-known phenomenon that perceiving an outcome as nearly (but ultimately not) occurring has powerful psychological consequences [43]–[45]. More specifically, almost attaining the desired outcome makes the counterfactual outcome (e.g., I could have won) more salient than when not having been close to the desired outcome [45]. This theory of counterfactual thinking may explain our results, as well as why, for example, Olympic silver medalist feel worse than bronze medalists: The silver medalists presumably have in mind they could have won the gold medal, whereas the bronze medalists are happy they won a medal at all [46].

### Behavioral Dynamics

The dynamics of the behavioral performance variable effort exertion were not characterized by straightforward upward or downward trends during positive and negative momentum. Strikingly, exerted efforts followed a pattern that has been proposed by Adler’s early social theory of momentum [1]. In the positive momentum session we found high exerted efforts at the start, which corresponds to the “building momentum” phase [1]. Subsequently, when winning two seconds efforts decreased and moved to a relatively stable exertion, which corresponds to a “cruising” phase [1]. Then a short significant drop in efforts occurred, which is in line with a coasting tendency [1], and which has also been reported in earlier research on individual PM [14]. Finally, effort exertion increased, which is in line with the “final kick” phase, reflecting a last boost in efforts when perceiving that the goal is near [1].

Negative momentum involved a steeper overall decrease in exerted efforts than positive momentum. Moreover, the effort exertion decreased over the entire negative momentum phase, which corresponds to a sinking tendency according to the early momentum theory [1]. The decrease in exerted efforts was rapid between time gap values of +6 s and +2 s, which could be interpreted as an early dropping tendency because of losing hope in a positive outcome (see also [23]). Noteworthy, two teams in our original sample showed an even more striking dropping tendency, these teams literally gave up when perceiving the opponent was coming back. This latter tendency supports that people sometimes voluntarily drop the activity when they reach a point at which they become certain that they will fail [1].

The second performance variable assumed to be involved in the team PM process was interpersonal coordination. Again in line with Adler’s theory of momentum, [1], we found that the quality of interpersonal coordination was higher during positive momentum than during negative momentum. Moreover, the stability of the coordination (relative phase) improved during positive momentum. We did, however, not find clear patterns with regard to the mean relative phase over the course of positive and negative momentum. The absence of such patterns could be explained by the robust finding that people automatically coordinate their movements over time when they are performing a comparable rhythmical task [47]–[53]. This continuous synchronization tendency could have been further strengthened by the fact that our sample consisted of rowing teams that were trained to stay coordinated.

## Conclusions and Future Directions

In conclusion, in the current article we introduced a dynamical approach to study the team PM process. We showed that, relative to positive team momentum, negative momentum elicits stronger (opposite) psychological changes and accompanies different (less adaptive) behavioral regulation. The asymmetry between positive and negative psychological team momentum dynamics, depending on the history of progress and regress, points to the relevance of pursuing a dynamical approach. Within the domain of social sciences–and team dynamics in particular–patterns of change often remain unnoticed, because optimal standardization and ruling out the role of history are common practice in mainstream experimental designs [25]. In addition, the results of exerted efforts and interpersonal coordination brought insights into the actual performance dynamics during positive and negative momentum. The lack of consistent results with regard to the momentum – performance outcome relationship in earlier research might be explained by our findings that performance processes are non-stationary during positive and negative momentum. Indeed, if we would have taken single snapshots of exerted force at some time-gap value in the positive or negative momentum session, for instance, we could have observed values reflecting relatively high, medium, and low performance.

Our results provide the first quantitative insights into the dynamical process of team PM. One may object that the sample size on which our insights are based is rather small. However, when studying processes, small samples can be very valuable provided that the cases (i.e., participants) are well-chosen [54]. In the current study, we selected competitive rowers for whom reaching a goal during an ergometer competition was meaningful. This selection was necessary to ensure that progressing and regressing in relation to that goal would elicit a positive and negative PM experience. Obviously, giving priority to a high quality sample often has consequences for the quantity of the sample.

Another point that should be noted is that the dynamical experimental method we applied is often used to find classical dynamical patterns in terms of stability and metastability [3], [4], [48], [51], [52], which we did not primarily focus on. Rather, we described and interpreted our results in terms of asymmetric and history-dependent patterns which, according to us, can be considered interesting dynamics underlying human psychological and behavioral functioning. Indeed, while the “classical” dynamical patterns are often found in physics and motor control, human psychological and behavioral systems could often be characterized by various dynamical trajectories [22]. Related to this, we may conclude that future researchers who aim to study psychological and behavioral processes would benefit from an approach that is specifically focused on describing and explaining change. A dynamical systems design as we applied (but also model simulations and dynamical research in natural situations, see [25], [39]) could greatly aid in getting a better grip on the dynamical nature of social and performance-related phenomena such as team psychological momentum.
